# Impact of Falls on Quality of Life among Filipinos with Parkinson’s Disease from a Referral Center Ambulatory Care PD Clinic

**DOI:** 10.31662/jmaj.2024-0144

**Published:** 2024-11-18

**Authors:** Jeryl Ritzi T. Yu, Anna Deborah L. Sarmiento-Santos, Milthon Cua, Michelle J. Tanglao, Raymond L. Rosales

**Affiliations:** 1 Movement Disorders Center, St. Luke’s Medical Center, Global City, Philippines; 2 Institute for Neurosciences, St. Luke’s Medical Center, Quezon City, Philippines; 3 University of the East, Ramon Magsaysay Memorial Medical Center, Quezon City, Philippines; 4 Taguig Pateros District Hospital, Taguig, Philippines; 5 R. De C Galvez Medical Center, Bulacan, Philippines; 6 Manila Doctors Hospital, Manila, Philippines; 7 Center for Neurodiagnostic and Therapeutic Services, Metropolitan Medical Center, Manila, Philippines; 8 Research Center for Health Sciences, University of Santo Tomas- FMS, Manila, Philippines

**Keywords:** Fall, Parkinson’s disease, Quality of life, Parkinson’s Disease Questionnaire 8

## Introduction

In 2020, Filipinos aged 60 years and above accounted for 9.2 million, and 1 in 500 people were found to have Parkinson’s disease (PD) ^(1)^. PD is the second most common neurodegenerative disorder worldwide. In the earlier stages of the disease, motor symptoms impair the patient’s ability to perform activities of daily living (ADLs). As PD advances, falls may complicate care and increase dependence on caregivers and are one of the most significant causes of disability in people with PD (PwP).

Studies have shown that 45%-68% of PwP fall each year, and up to 50%-86% of the population experience recurrent falls ^(2)^. PwP were also noted to have increased risk of falls and fractures at the age of 40, which is earlier compared with that of healthy individuals. As a consequence, the individual develops fear of falling, which limits the performance of ADLs, resulting in high levels of caregiver stress and dependence that greatly affect quality of life (QoL).

One of the major determinants of QoL is safety, which is compromised in PwP due to their motor symptoms. They are more prone to accidents such as falls, rendering them vulnerable and unable to perform ADLs, ultimately affecting their perception of QoL. In this study, we aimed to compare the QoL among PwP with and without falls in a Filipino cohort.

## Methods

This is a single-center retrospective study, including all patients who were clinically diagnosed with PD using the UK Parkinson’s Disease Society Brain Bank Criteria by a movement disorder specialist (RR), with clinic visits between January 2021 and December 2021. Patients 18 years old and above, diagnosed with PD, and treated with either monotherapy or combination therapy were included. The following diagnoses were excluded to minimize heterogeneity of the cohort: atypical parkinsonism (progressive supranuclear palsy, multiple system atrophy, Lewy body dementia, or corticobasal syndrome), vascular parkinsonism, normal pressure hydrocephalus, and secondary drug-induced parkinsonism. These conditions may present with more progressive symptoms (e.g., orthostatic hypotension, postural instability, hallucinations, etc.) earlier on in the disease course compared with typical PD, which may increase risk of falls, and hence excluded to minimize heterogeneity of the cohort. Using a confidence interval of 95%, the computed sample size is at least 74.

JY, AS, and MT reviewed medical records. Any fall within the past 3 months was included. Falls were defined as “any unexpected event that caused the person to unintentionally land on any lower surface (object, floor, or ground)” ^(3)^. The following demographic and clinical data were obtained: age, gender, duration since formal diagnosis, levodopa equivalent daily doses (LEDD) in mg, presence or absence of at least one episode of fall over the past 3 months, postural instability, freezing of gait (FOG), location of falls, and area of injury for those who sustained falls, motor evaluation (UPDRS III), motor complications (dyskinesias and on-off fluctuations) from UPDRS IV, and Hoehn and Yahr (H&Y) scores. To assess the patient’s QoL, we used the PDQ-8 ^(4)^ and responses were presented using tables and proportions of responses per category. T-test and two proportion z-test under 95% CI level were used to determine whether there was significant difference between variables. Data were analyzed using SPSS software version 29. This study was approved by the UERM RIHS Ethics Review Committee, number 1230/E/2022/040.

## Results

A total of 78 individuals with PD were included in this study (Table 1). The mean age at assessment was 70.51 ± 12.82, and most were females (n = 46, 58.97%). Most had a PD duration of 0-10 years (n = 54, 69.23%). Over half had combination therapy of medications (n = 40, 51.28%) and exhibited postural instability (n = 48, 61.54%), whereas 25 patients (32.05%) demonstrated FOG. A total of 15 patients (19.23%) reported episodes of falls, most of which occurred in the home setting (n = 12, 80%). All 15 PwP with episodes of falls had at least one of or both postural instability and FOG.

**Table 1.** Demographics and Clinical Data of Patients.

	Total (n = 78, 100%)
Age ≤60 years old 61 to 65 years old 66 to 70 years old 71 to 75 years old 76 to 80 years old >80 years old	Mean (± SD): 70.51 ± 12.82 16 (20.51%) 8 (10.26%) 11 (14.10%) 11 (14.10%) 14 (17.95%) 18 (23.08%)
Sex Male Female	32 (41.03%) 46 (58.97%)
Duration of Parkinson’s disease 0-10 years 11-20 years ≥21 years	54 (69.23%) 22 (28.21%) 2 (2.56%)
Medications Monotherapy Combination therapy	38 (48.72%) 40 (51.28%)
Episodes of falls Yes No	15 (19.23%) 63 (80.77%)
Location of falls (n = 15) Home Outside home Hospital	12 (80%) 2 (13.33%) 1 (6.67%)
Area of injury (n = 15) Limb Head Limb and trunk	6 (40%) 6 (40%) 3 (20%)
Postural instability Yes No	48 (61.54%) 30 (38.46%)
Freezing gait Yes No	25 (32.05%) 53 (67.95%)

The population’s responses to PDQ-8 were grouped to those with and without episodes of falls (Table 2). Overall, PwP without episodes of falls had the least “often” or “always” answers to questions on difficulties and challenges such as “Had difficulty getting around in public?” and “Had difficulty dressing yourself?”, as compared with those with episodes of falls. The mean PDQ-8 score of those with falls was significantly higher than that of those without (13.00 ± 4.74 vs 6.32 ± 4.92 respectively, p < 0.00001).

**Table 2.** Patient Responses to PDQ-8 Questionnaire Regarding Quality of Life.

	Total (n = 78, 100%)	Patients with falls (n = 15)	Patients without falls (n = 63)
Had difficulty getting around in public? Never Occasionally Sometimes Often Always	4 (5.13%) 6 (7.69%) 16 (20.51%) 24 (30.77%) 28 (35.90%)	0 (0%) 0 (0%) 0 (0%) 3 (20%) 12 (80%)	4 (6.35%) 6 (9.52%) 16 (25.40%) 21 (33.33%) 16 (25.40%)
Had difficulty dressing yourself? Never Occasionally Sometimes Often Always	12 (41.03%) 14 (17.95%) 17 (21.79%) 8 (10.26%) 7 (8.97%)	1 (6.67%) 2 (13.33%) 1 (6.67%) 6 (40%) 5 (33.33%)	31 (49.21%) 12 (19.05%) 16 (25.40%) 2 (3.17%) 2 (3.17%)
Felt depressed? Never Occasionally Sometimes Often Always	50 (64.10%) 9 (11.54%) 14 (17.95%) 4 (5.13%) 1 (1.28%)	7 (46.67%) 2 (13.33%) 2 (13.33%) 4 (26.67%) 0 (0%)	43 (68.25%) 7 (11.11%) 12 (19.05%) 0 (0%) 1 (1.59%)
Had problems with your close personal relationships? Never Occasionally Sometimes Often Always	60 (76.92%) 5 (6.41%) 8 (10.26%) 3 (3.85%) 2 (2.56%)	8 (53.33%) 0 (0%) 5 (33.33%) 2 (13.33%) 0 (0%)	52 (82.54%) 5 (7.94%) 3 (4.76%) 1 (1.59%) 2 (3.17%)
Had problems with your concentration, e.g., when reading or watching TV? Never Occasionally Sometimes Often Always	56 (71.79%) 12 (15.38%) 9 (11.54%) 1 (1.28%) 0 (0%)	6 (40%) 4 (26.67%) 4 (26.67%) 1 (6.67%) 0 (0%)	50 (79.37%) 8 (12.70%) 5 (7.94%) 0 (0%) 0 (0%)
Felt unable to communicate with people properly? Never Occasionally Sometimes Often Always	48 (61.54%) 16 (20.51%) 10 (12.82%) 3 (3.85%) 1 (1.28%)	6 (40%) 3 (20%) 3 (20%) 2 (13.33%) 1 (6.67%)	42 (66.67%) 13 (20.63%) 7 (11.11%) 1 (1.59%) 0 (0%)
Had painful muscle cramps or spasms? Never Occasionally Sometimes Often Always	41 (52.56%) 12 (15.38%) 19 (24.36%) 5 (6.41%) 1 (1.28%)	2 (13.33%) 3 (20%) 7 (46.67%) 3 (20%) 0 (0%)	39 (61.90%) 9 (14.29%) 12 (19.05%) 2 (3.17%) 1 (1.59%)
Felt embarrassed in public due to having Parkinson’s disease? Never Occasionally Sometimes Often Always	64 (82.05%) 5 (6.41%) 3 (3.85%) 6 (7.69%) 0 (0%)	14 (93.33%) 0 (0%) 1 (6.67%) 0 (0%) 0 (0%)	50 (79.37%) 5 (7.94%) 2 (3.17%) 6 (9.52%) 0 (0%)

The mean (±SD) of the UPDRS III score among all participants was 34.9 ± 16.1. Patients with falls had significantly higher scores than those without, with means (±SD) of 54.53 ± 13.66 and 30.19 ± 12.99, respectively (p < 0.00001). The mean (±SD) H&Y score among those with falls was also significantly higher than those without (3.40 ± 1.11 and 2.42 ± 0.82, respectively; p < 0.0001). The mean age and mean duration with PD were also significantly higher among those with falls than those without (80.60 ± 7.75 vs 68.11 ± 12.65; p = 0.0002 and 12.73 ± 4.50 vs 7.50 ± 5.82; p = 0.0008, respectively) (Table 3). There were no significant differences in UPDRS IV, mean LEDD scores, and gender ratios. Those with episodes of falls exhibited a higher percentage in both FOG (n = 11, 73.7%) and postural instability (n = 15, 100%) than those without, with p-values of <0.00007 and <0.0003, respectively.

**Table 3.** Comparison of PDQ-8, UPDRS III, UPDRS IV, LEDD, H&Y Scores, Mean Age, Sex, and PD Duration between Patients with and without Episodes of Falls.

	Patients with episodes of falls (n = 15)	Patients without episodes of falls (n = 63)	p-value
PDQ-8 score	13.00 ± 4.74	6.32 ± 4.92	<0.00001
UPDRS III score	54.53 ± 13.66	30.19 ± 12.99	<0.00001
UPDRS IV score	4.07 ± 1.53	4.13 ± 2.00	0.4567
Levodopa equivalent daily dose (in mg)	568.87 ± 283.74	499.07 ± 384.48	0.2556
Mean Hoehn and Yahr score	3.40 ± 1.11	2.42 ± 0.82	0.0001
Mean age in years	80.60 ± 7.75	68.11 ± 12.65	0.0002
Sex: Male Female Female/male ratio	40% (n = 6) 60% (n = 9) 1.5000	41.27% (n = 26) 58.73% (n = 37) 1.4231	0.4641
PD duration in years	12.73 ± 4.50	7.50 ± 5.82	0.0008

## Discussion

Compared with large volume data from the Parkinson’s Foundation registry, our PD ambulatory care cohort had a higher proportion of PwP with falls at 19% compared with their results (11.7%). In a large-scale survey that examined the QoL based on PDQ-39 among PwP, fear of falling was estimated to be present in 37%-59% of PwPs and accounted for limitations in activity in 70% ^(5)^. These findings are supported by other studies that demonstrated that fear of falling is an independent risk factor of falling ^(6)^ and a history of prior fall was one of the strongest predictors ^(7)^, reducing QoL.

Other factors that have been shown to increase risk of falls include increased disease severity and impaired balance ^(8)^. This is supported by our data showing significantly higher mean (±SD) UPDRS III and H&Y scores of PwPs with falls than those without. In addition, our data showed that most (86.6%) of those with falls had at least some postural instability denoted by an H&Y score of at least 3. Among PwP with falls, 73.33% experienced FOG, and all were found to have postural instability. These findings align with a previous study where unstable posture, FOG, and sudden loss of postural reflexes were identified as the most common causes of falls among PwPs ^(9)^.

These results highlight the significance of engaging in exercises led by a PD-trained physical therapist to minimize FOG. Clinicians should also identify whether FOG occurs during the on- or off-state to guide optimization of the patient’s levodopa regimen. Other strategies to overcome FOG (e.g., cueing, etc.) have also been helpful in many patients and may benefit from practicing these to reduce falls and improve QoL. Virtually all individuals with episodes of falls experienced postural instability. This implies that QoL is reduced when balance and postural reflexes are compromised in PD. Therefore, it is crucial to engage in balance training and to ensure that safety measures (e.g., installation of handrails, non-slip mats, decluttering walkways, etc.) in the patient’s natural environment are in place.

Although all patients in this cohort who reported falls had postural instability, studies have shown that certain groups of patients experience falls despite absence of postural instability ^(10)^, further supporting the multifactorial nature of falls. Aside from gait changes and motor impairment, orthostatic hypotension, which is experienced by up to 40% of PwP ^(11)^, is a critical contributory factor to falls. Although falls may also be related to dyskinesias and total levodopa dose, there was no statistically significant difference between UPDRS IV and LEDD between those with and without falls.

Among the PDQ-8 questions, “difficulty getting around in public” was consistently rated “often” and “always” by both groups. All PwPs with falls rated it either “often” or “always,” whereas over half of PwPs without falls rated them “often” or “always.” The mean PDQ-8 scores of those with falls were significantly higher than those without, denoting a poorer QoL among those with falls. Interestingly, 80% of PwP with episodes of falls experienced falls in the home setting, and only one had an in-hospital fall. These data are notable as hospitalized patients are at an increased risk for falls ^(12)^. One possible reason for falls occurring mostly at home is that this cohort was assessed during the pandemic when individuals still chose to stay home. In addition, this study was conducted among a Filipino cohort in the outpatient setting, most of whom have a care partner (family member or private nurse) and reside in their personal home setting rather than in a nursing home/hospice setting.

This study has some limitations. Given the retrospective nature of this study, only presence or absence of episodes of falls was available. An analysis of the frequency and severity of falls may have yielded additional information and provided deeper insights into the impact of falls on the patients’ QoL. In addition, the sample size was relatively small with a small proportion of falls, which may present statistical bias. Most individuals with falls presented with one or both of postural instability and FOG. Other contributory factors such as cognitive decline and fall-related comorbidities may also increase the risk for falls. Inclusion of these data, if available, would have allowed a better understanding of the circumstances of falls. Lastly, although we made efforts to include individuals with PD in the early and late stages of the disease, we recognize that the results may not entirely be generalizable to larger populations as the study was conducted only in a single referral center. Future studies may consider a longitudinal design to allow better monitoring of patients and better understanding of causative factors. A multicenter study would also allow better generalizability to other settings and populations.

In conclusion, PwP with falls obtained higher UPDRS III scores and experienced FOG and postural instability compared with PwP without falls. They also had higher PDQ-8 scores than those without falls, denoting a poorer QoL. This highlights the significance of education and practice of safety measures to prevent falls, especially among those with FOG and postural instability, as they lead to a significantly poorer QoL.

## Article Information

### Conflicts of Interest

None

### Author Contributions

Conceptualization: RR. Data curation: JY, AS, and MT. Formal analysis: MC. Methodology: JY, AS, MC, and RR. Project administration: JY, AS, MT, and MC. Supervision: RR. Writing―original draft: JY and AS. Writing―review and editing: all authors

### Approval by Institutional Review Board (IRB)

This study was approved by the UERM RIHS Ethics Review Committee, number 1230/E/2022/040. A written consent was obtained from all participants.
